# ETV4 collaborates with Wnt/β-catenin signaling to alter cell cycle activity and promote tumor aggressiveness in gastrointestinal stromal tumor

**DOI:** 10.18632/oncotarget.23173

**Published:** 2017-12-11

**Authors:** Shan Zeng, Adrian M. Seifert, Jennifer Q. Zhang, Teresa S. Kim, Timothy G. Bowler, Michael J. Cavnar, Benjamin D. Medina, Gerardo A. Vitiello, Ferdinand Rossi, Jennifer K. Loo, Nesteene J. Param, Ronald P. DeMatteo

**Affiliations:** ^1^ Department of Surgery, Memorial Sloan Kettering Cancer Center, New York, NY, USA; ^2^ Department of Medicine, Memorial Sloan Kettering Cancer Center, New York, NY, USA

**Keywords:** gastrointestinal stromal tumor, ETV4, Wnt/β-catenin signaling

## Abstract

Gastrointestinal stromal tumor (GIST) is the most common sarcoma, often resulting from a *KIT* or platelet-derived growth factor receptor alpha (*PDGFRA*) mutation. The lineage transcription factor ETV1 is expressed similarly in GISTs regardless of malignant potential. Although the related transcription factor ETV4 has been associated with metastasis and tumor progression in other cancers, its role in GIST is unknown. In this study, we found that ETV4 levels were high in a subset of human GISTs and correlated with high mitotic rate. Through Gene Set Enrichment Analysis in selected human GISTs, we identified a relationship between ETV4 levels and β-catenin signaling, especially in advanced GISTs. GIST specimens with high ETV4 levels overexpressed cell cycle regulating genes and had aberrant activation of the canonical Wnt pathway. In human GIST cell lines, ETV4 RNA interference suppressed cell cycle genes and Wnt/β-catenin signaling. ETV4 knockdown also reduced tumor cell proliferation, invasion, and tumor growth *in vivo*. Conversely, ETV4 overexpression increased cyclin D1 expression and Wnt/β-catenin signaling. Moreover, we determined that ETV4 knockdown destabilized nuclear β-catenin and increased its degradation via COP1, an E3 ligase involved in both ETV4 and β-catenin turnover. Aberrant accumulation of ETV4 and nuclear β-catenin was found in patient derived xenografts created from metastatic GISTs that became resistant to tyrosine kinase inhibitors. Collectively, our findings highlight the significance of ETV4 expression in GIST and identify ETV4 as a biomarker in human GISTs.

## INTRODUCTION

Gastrointestinal stromal tumor (GIST) is the most common subtype of human sarcoma and typically occurs in the stomach or small intestine [1]. The majority of GISTs are driven by an activating mutation in either *KIT* or *PDGFRA* [2, 3]. The selective tyrosine kinase inhibitor imatinib has been used as the standard therapy for GIST and dramatically improved survival [4]. Unfortunately, imatinib is rarely curative, and resistance commonly occurs within 2 years of treatment, often via a secondary *KIT* mutation [5]. Although alternative tyrosine kinase inhibitors can overcome imatinib resistance temporarily [6, 7], the vast majority of patients with metastatic GIST develop tumor progression and eventually die. The mechanisms underlying GIST aggressiveness are only partially defined. While *p16* (*CDKN2A*) gene deletion and inactivation of myc-associated protein (MAX) are found to be common genetic aberrations in GIST progression [8, 9], a better understanding of the molecular mechanisms responsible for GIST aggressiveness may identify clinical biomarkers or new therapeutic targets.

ETV1, ETV4, ETV5, and ERG are members of the erythroblast transformation specific (ETS) transcription factor family and are well established as oncogenes in certain cancers [10, 11], while ETV6 gene fusions have been reported in GIST [12]. Although they share highly conserved DNA-binding domains, their different roles and functions are not precisely known. ETV1 has been identified as a lineage survival factor in GIST and a master regulator in controlling development and hyperplasia of the interstitial cells of Cajal (ICC) [13]. Targeting ETV1 stability inhibited GIST tumorigenicity [14]. However, the magnitude of ETV1 expression by immunohistochemistry did not correlate with progression in patients with GIST [15]. Previously, we found that ETV4 transcription was significantly reduced by imatinib treatment and ETV4 modulated the transcription of the immunosuppressive enzyme indoleamine 2,3-dioxygenase (IDO) [16]. Intriguingly, cooperative relationships have been described between ETS family members, receptor tyrosine kinases, and alternative signaling pathways, resulting in aggressive phenotypes. For example, combined expression of ERG with additional genetic alterations can lead to malignancy in prostate cancer [17]. Moreover, ETV4 promoted metastasis in response to PI3K/AKT activation [18].

The role of ETV4 in GIST has not been defined. In this study, we discovered that ETV4 was overexpressed in a subset of human GISTs and modulated cell cycle regulation and Wnt/β-catenin signaling, which we previously demonstrated to be important in GIST malignancy [19]. Knockdown of ETV4 suppressed cell proliferation, tumor invasion, and growth. In addition, silencing of ETV4 destabilized nuclear β-catenin by increasing its interaction with COP1, resulting in β-catenin proteasomal degradation. Therefore, ETV4 collaborates with Wnt/β-catenin signaling to potentiate GIST aggressiveness.

## RESULTS

### ETV4 is highly expressed in a subset of aggressive human GISTs

**Figure 1:**
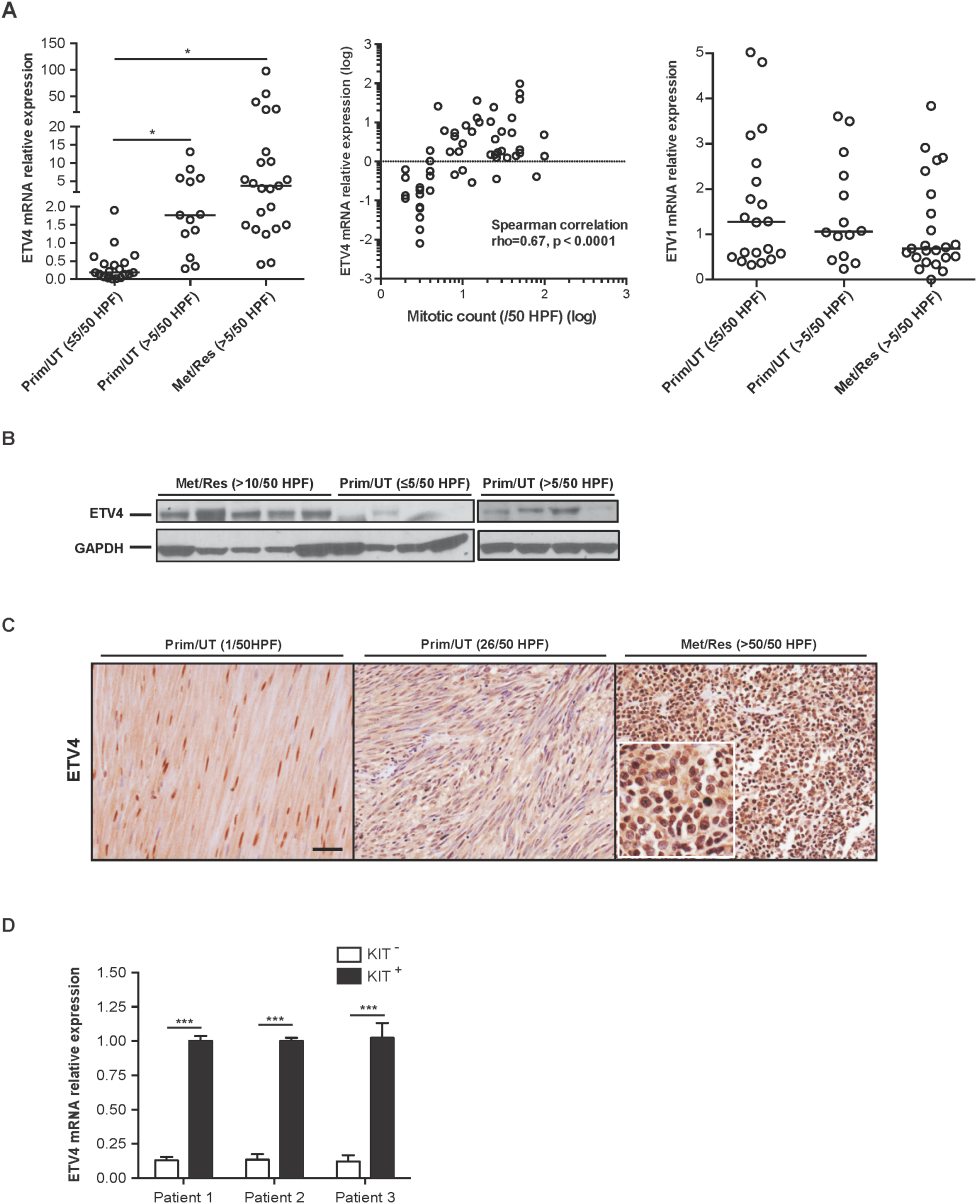
ETV4 is highly expressed in a subset of aggressive human GISTs **(A)** RNA was isolated from 55 freshly frozen human GISTs and analyzed for ETV4 and ETV1 using real-time PCR. Prim/UT–primary, untreated GIST, Met/Res – metastatic, imatinib-resistant GIST, HPF – high power field. Horizontal bars represent the median (left and right). Student’s *t* test, ^*^P < 0.05. A scatter plot shows the correlation between ETV4 and mitotic count (middle) (Spearman’s rho = 0.67 with *P* = 0.001 per 2-tailed test). **(B)** Protein extracts were prepared from freshly frozen human GISTs that were either primary, untreated tumors with a mitotic rate of ≤5/50 HPF or >5/50 HPF, and metastatic, imatinib-resistant GISTs with a mitotic rate of >10/50 HPF and immunoblotted with anti-human ETV4 IgG followed by anti-GAPDH IgG. **(C)** Representative ETV4 staining from 46 paraffin-embedded human GISTs. Scale bar, 20 μm. Inset is 40x magnification to show nuclear staining. **(D)** Freshly isolated KIT^-^ and KIT^+^ cells from 3 metastatic, imatinib-resistant human GISTs with high ETV4 expression were analyzed for ETV4 mRNA by real-time PCR. Bars, mean ± SEM. Student’s *t* test, ^***^P <0.001.

To evaluate the importance of ETV4 in GIST biology, we first examined ETV4 mRNA expression by real-time PCR in 55 freshly frozen human GIST surgical specimens (Supplementary Table 1). ETV4 mRNA expression correlated with tumor mitotic rate in primary and metastatic GIST (Figure 1A, left and middle). Most untreated, primary GISTs with a low mitotic rate had low or undetectable levels of ETV4 expression while those with a high mitotic rate generally had high ETV4 expression. Similarly, ETV4 was upregulated in a substantial proportion of metastatic GISTs that were resistant (i.e., progressed on tyrosine kinase inhibitors) and had a high mitotic rate (*P* < 0.05). Conversely, expression of the GIST lineage survival factor ETV1 did not correlate with mitotic rate or whether a tumor was metastatic (Figure 1A, right), although the magnitude of ETV1 mRNA expression was much greater than that of ETV4. Next, we validated the ETV4 expression levels in selected ETV4-high (metastatic, imatinib-resistant GISTs with a mitotic rate >10/50 HPF) and ETV4-low tumors (untreated, primary GISTs with a mitotic rate ≤5/50 or >5/50 HPF) by western blot (Figure 1B). By immunohistochemistry, ETV4 localized to the nucleus, and was more prevalent in tumors with high mitotic rates and was less frequent in tumors with low mitotic rates (Figure 1C). After freshly isolating KIT^+^ and KIT^-^ cells from 3 human metastatic, imatinib-resistant GISTs with high ETV4 expression, we showed that ETV4 mRNA was minimal in KIT^-^ (i.e., non-tumor) cells (Figure 1D). Taken together, ETV4 was overexpressed in human GISTs, especially those with a high mitotic rate, which is an established indicator of aggressive biology in GIST [20].

### ETV4 knockdown reduces tumor cell proliferation, tumor invasion, and growth

**Figure 2:**
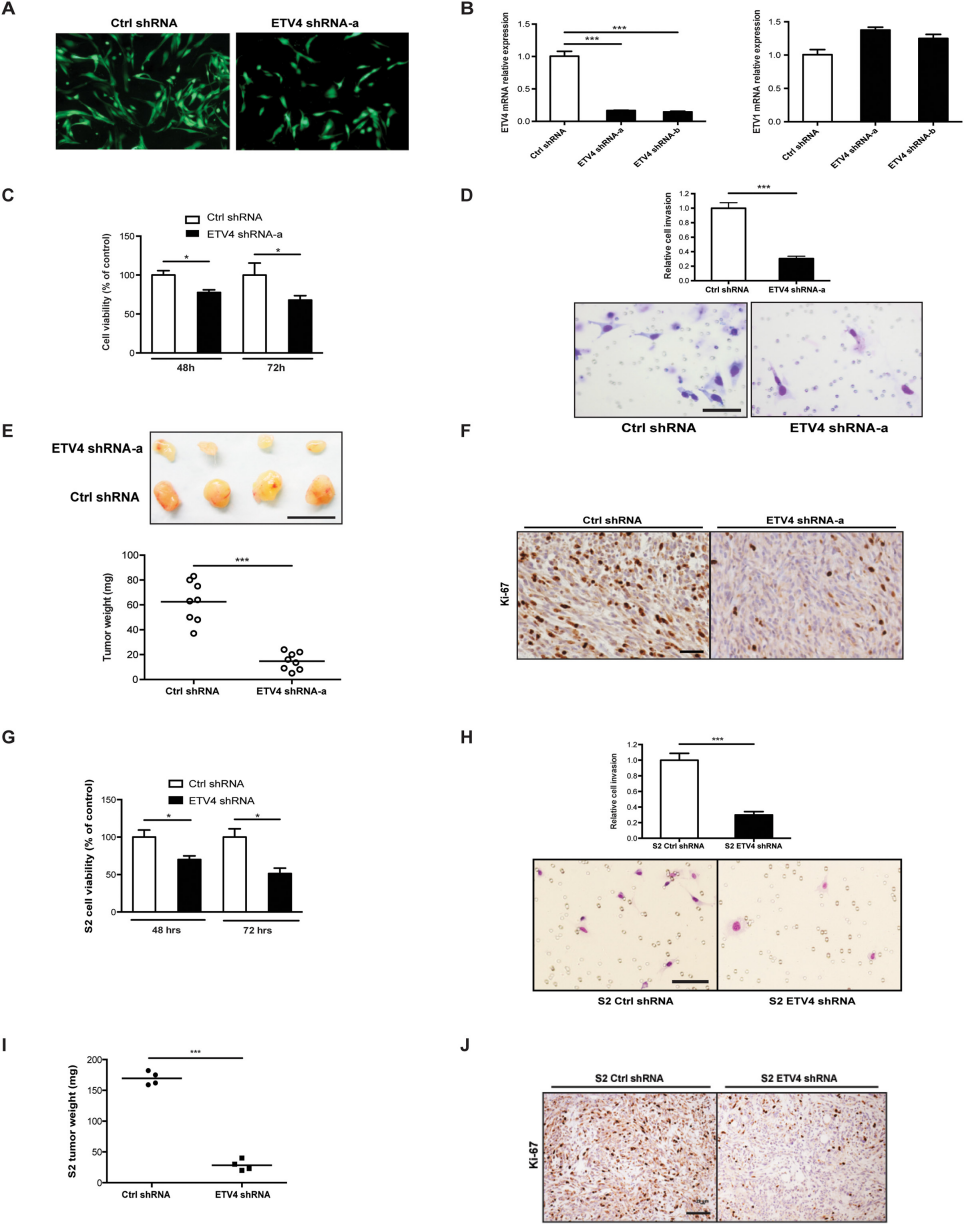
ETV4 knockdown reduces tumor cell proliferation, tumor invasion, and growth **(A)** GFP expression in GIST882 cells after transduction with either ETV4 or control shRNA lentiviral constructs and growth under continuous puromycin selection. **(B)** ETV4 and ETV1 mRNA expression measured by real-time PCR. **(C)** Cell viability of GIST882 cells after ETV4 knockdown. **(D)** Matrigel invasion assay of GIST882 cells after ETV4 knockdown. Scale bar, 100 μm. **(E)** 1x10^5^ GIST882 cells stably transduced with ETV4 or control shRNA were injected into the flanks of NSG mice and tumors were harvested 4 months later. Representative gross pictures and tumor weights are shown. n = 8 mice per group. Scale bar, 1 cm. **(F)** Ki-67 staining in ETV4 knockdown or control tumors. Scale bar, 20 μm. **(G)** Cell viability of murine S2 cells after stable ETV4 knockdown by shRNA infection. **(H)** Matrigel invasion assay of murine S2 cells after ETV4 knockdown. **(I)** Weights of flank tumors in NSG mice 4 months after inoculation with 1x10^5^ S2 cells transduced with ETV4 or control shRNA (n = 4 mice per group). **(J)** Representative Ki-67 staining in ETV4 knockdown or control S2 tumors. Scale bar, 20 μm. Lines represent the median. All bars, mean ± SEM, Student’s *t* test; ^*^P < 0.05, ^***^P < 0.001.

To understand the functional significance of ETV4 expression in GIST, we first established GIST882 cell lines with either stably transduced ETV4 shRNA or scrambled shRNA. Stable cell lines were established when all cells expressed GFP under continuous puromycin selection (Figure 2A). ETV4 knockdown was confirmed by real-time PCR, and importantly, ETV1 expression was unaffected (Figure 2B). ETV4 knockdown in GIST882 cells reduced tumor cell viability (Figure 2C) and invasion (Figure 2D) *in vitro*. In mouse flank tumor xenografts, ETV4 silencing also inhibited GIST882 tumor growth (Figure 2E), as well as tumor proliferation by Ki-67 staining (Figure 2F). We further confirmed these findings in the murine GIST cell line S2, which we previously derived from a *Kit*^*V558Δ/+*^ mouse that develops a single intestinal GIST [21]. As with GIST882 cells, ETV4 knockdown in murine S2 GIST cells reduced tumor cell viability (Figure 2G) and invasion *in vitro* (Figure 2H), as well as tumor growth and Ki-67 staining *in vivo* (Figure 2I-2J). In fact, there was a greater than 6-fold reduction in tumor size in ETV4 silenced S2 cells compared to control cells, which is substantial for the S2 GIST cell line. Taken together, ETV4 regulated tumor cell growth both *in vitro* and *in vivo*.

### Knockdown of ETV4 modulates cell cycle genes and Wnt signaling in GIST cell lines

**Figure 3:**
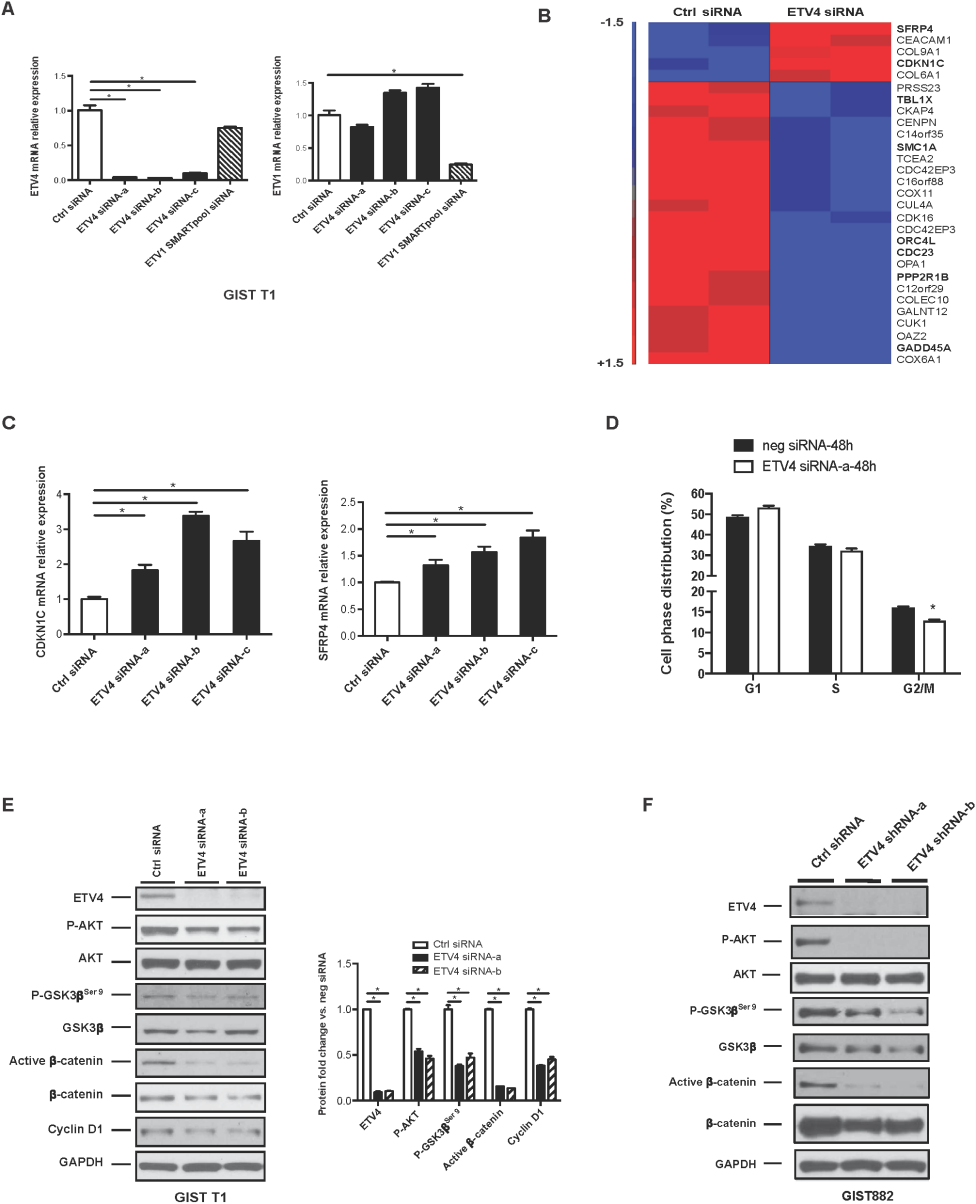
Knockdown of ETV4 modulates cell cycle genes and Wnt signaling in GIST cell lines **(A)** Human GIST T1 cells transfected with ETV4 siRNA, control siRNA, or ETV1 SMARTpool siRNA were assayed for ETV4 or ETV1 expression by real-time PCR. **(B)** Transcriptome analysis of GIST T1 cells following transfection with either ETV4 siRNA or control siRNA for 48h (n=2/group). **(C)** Real-time PCR for CDKN1C and SFRP4 mRNA. **(D)** Analysis of cell cycle profile in GIST T1 cells after 48h transfection with control siRNA or ETV4 siRNA. **(E)** Western blot of GIST T1 cells following transfection with either ETV4 or control siRNA for 96h. Bar graph shows densitometry analysis of the relative expression normalized to GAPDH when compared to control (neg) siRNA. **(F)** Western blot of GIST882 cells following stable transduction with either ETV4 shRNA or control shRNA. All bars, mean ± SEM. Student’s *t* test; ^*^P < 0.05.

To further elucidate the mechanism of ETV4 on GIST tumorigenicity, we performed transcriptome profiling in GIST T1 cells (which have higher ETV4 expression levels than GIST882 cells) with either ETV4-specific siRNA or non-target control siRNA. Effective knockdown of ETV4 with different constructs was confirmed by real-time PCR (Figure 3A). Notably, ETV1 and ETV4 expression were independent, as knockdown of one did not decrease expression of the other. There were 182 genes with a False Discovery Rate of *P* <0.05 and a fold change >1.5 when comparing ETV4-specific siRNA knockdown to a control siRNA (Figure 3B). Using KEGG pathway analysis, we found that DNA replication and cell cycle genes were overrepresented (Supplementary Table 2), consistent with the association we had observed between ETV4 and mitotic rate (Figure 1). Silencing of ETV4 increased the relative expression of cyclin-dependent kinase inhibitor 1C (CDKN1C), a negative regulator of G1 cyclin/Cdk complexes and cell proliferation. Moreover, ETV4 knockdown inhibited the Wnt/β-catenin signaling pathway, which we recently identified to contribute to tumor malignancy in GIST [19]. Specifically, ETV4 knockdown upregulated secreted frizzled-related protein 4 (SFRP4), which acts as a negative modulator of canonical Wnt signaling. Loss of SFRP4 expression has been linked to tumor progression in pancreatic tumors [22]. We validated the CDKN1C and SFRP4 findings by performing real-time PCR with different constructs of ETV4 siRNA (Figure 3C). We also performed cell cycle analysis by FACS in control and ETV4 siRNA transfected cells to quantify cell cycle activity. We found that transient knockdown of ETV4 interrupted the cell phase distribution, especially G2/M phase (Figure 3D).

To extend our observations, we examined cell cycle and Wnt signaling components in GIST T1 cells after 96h of transfection using control siRNA or two ETV4 siRNA constructs. We found that ETV4 knockdown reduced the phosphorylation of AKT and GSK3β serine 9 in GIST T1 cells (Figure 3E), which facilitates formation of the β-catenin destruction complex [23]. Accordingly, ETV4 knockdown reduced active β-catenin, which was specifically detected by dephosphorylated Ser37 and Thr41 [24], as well as cyclin D1 protein (Figure 3E). Similarly, stable ETV4 knockdown by shRNA interference in GIST882 cells also significantly reduced the phosphorylation of AKT, GSK3β serine 9, active β-catenin, and total β-catenin compared to control shRNA (Figure 3F). Thus, ETV4 modulated the cell cycle and Wnt/β-catenin signaling in GIST cell lines.

### ETV4 overexpression increases cyclin D1 expression and Wnt/β-catenin signaling

**Figure 4:**
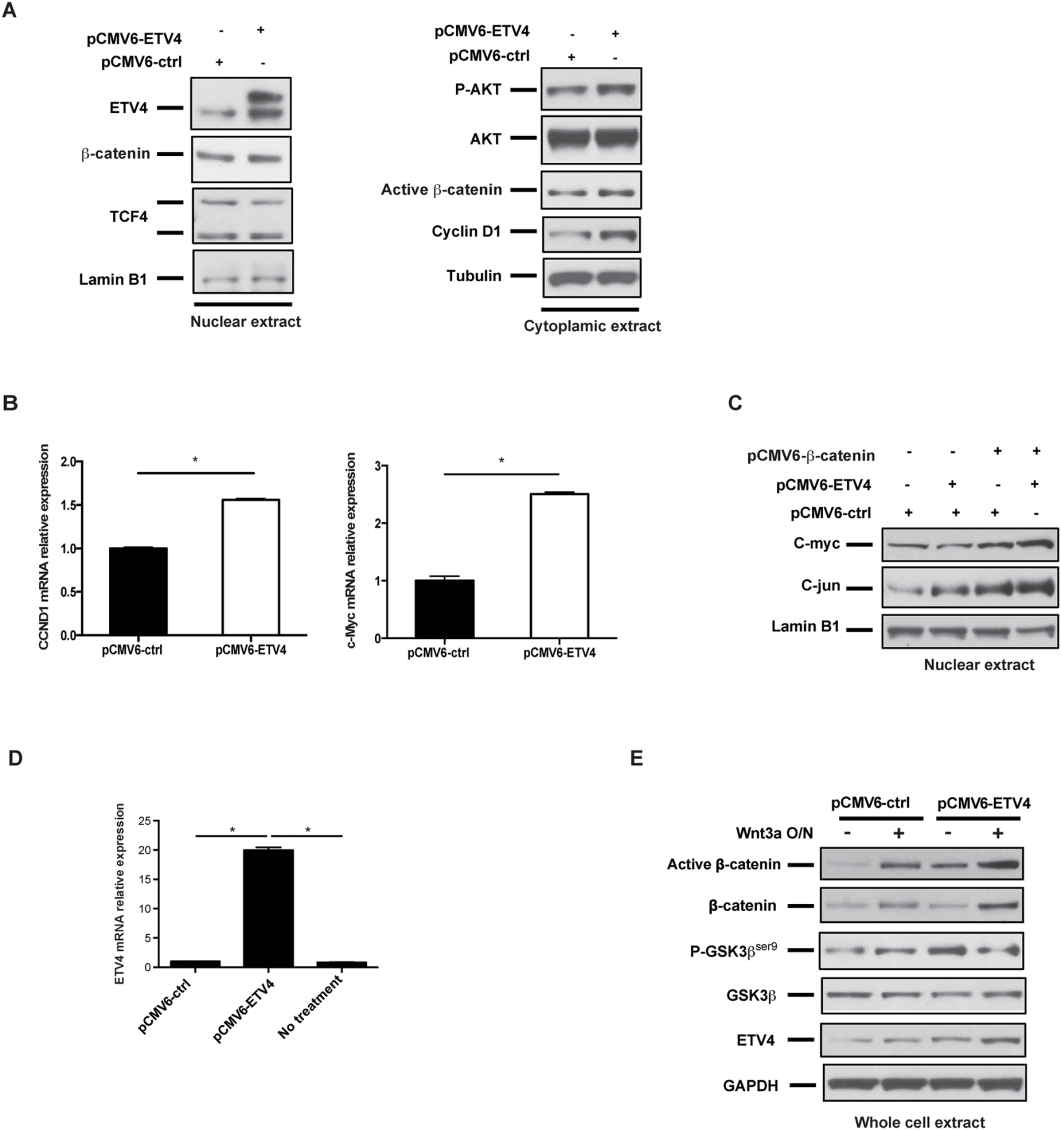
ETV4 overexpression increases cyclin D1 expression and Wnt/β-catenin signaling **(A)** Western blot of nuclear and cytoplasmic extracts from GIST T1 cells that had been transfected with control or ETV4 expression plasmid for 48h. **(B)** Real-time PCR of CCND1 (cyclin D1) and Myc mRNA expression in GIST T1 cells after 48h transfection with control or ETV4 expression plasmid. **(C)** GIST T1 cells were transfected with the indicated constructs for 48h. The nuclear extracts were used for western blot. **(D)** Real time PCR of ETV4 mRNA expression in GIST882 cells after 48h transfection with control or ETV4 expression plasmid. **(E)** GIST882 cells were transfected with either control or human ETV4 plasmids for 48h, and then cells were treated with or without 125 ng/ml rhWnt3a overnight. Total protein extracts were analyzed by western blot as indicated. All bars, mean ± SEM. Student’s *t* test; ^*^P < 0.05.

To test whether overexpression of ETV4 affects the cell cycle and Wnt/β-catenin signaling, we transfected GIST T1 cells with a control or an ETV4-overexpression plasmid. ETV4 overexpression was confirmed in the nuclear extract (Figure 4A, left), and as expected, ETV4 overexpression significantly increased the phosphorylation of AKT, cytoplasmic active β-catenin, and cyclin D1 expression (Figure 4A, right). ETV4 overexpression was also accompanied by the upregulation of cyclin D1 and c-Myc mRNA expression (Figure 4B). ETS family members have been reported to enhance promoter responsiveness to c-jun [25] and c-jun can stabilize β-catenin via its association with T cell factor 4 (TCF4) [26]. Thus, we co-transfected GIST T1 cells with β-catenin and ETV4 plasmids to determine their effects in combination. We found that upregulation of nuclear c-myc and c-jun protein was synergistically enhanced in ETV4 and β-catenin co-overexpressing cells (Figure 4C). To test whether ETV4 modulation also affects Wnt signaling during the Wnt-on state, we stimulated GIST882 cells that had been transfected with a control or an ETV4-overexpression plasmid in the presence or absence of the Wnt ligand Wnt3a. Although GIST882 cells had a relatively lower endogenous level of ETV4 compared to GIST T1 cells, ETV4-overexpression led to a 20-fold increase in ETV4 mRNA (Figure 4D). We found that the level of active β-catenin was significantly increased by Wnt3a stimulation and ETV4 overexpression individually, and even more so in combination (Figure 4E). These findings suggested that high levels of ETV4 might collaborate with Wnt/β-catenin signaling to promote tumor malignancy in GIST cells.

### ETV4 interacts with COP1 to regulate nuclear β-catenin stability

**Figure 5:**
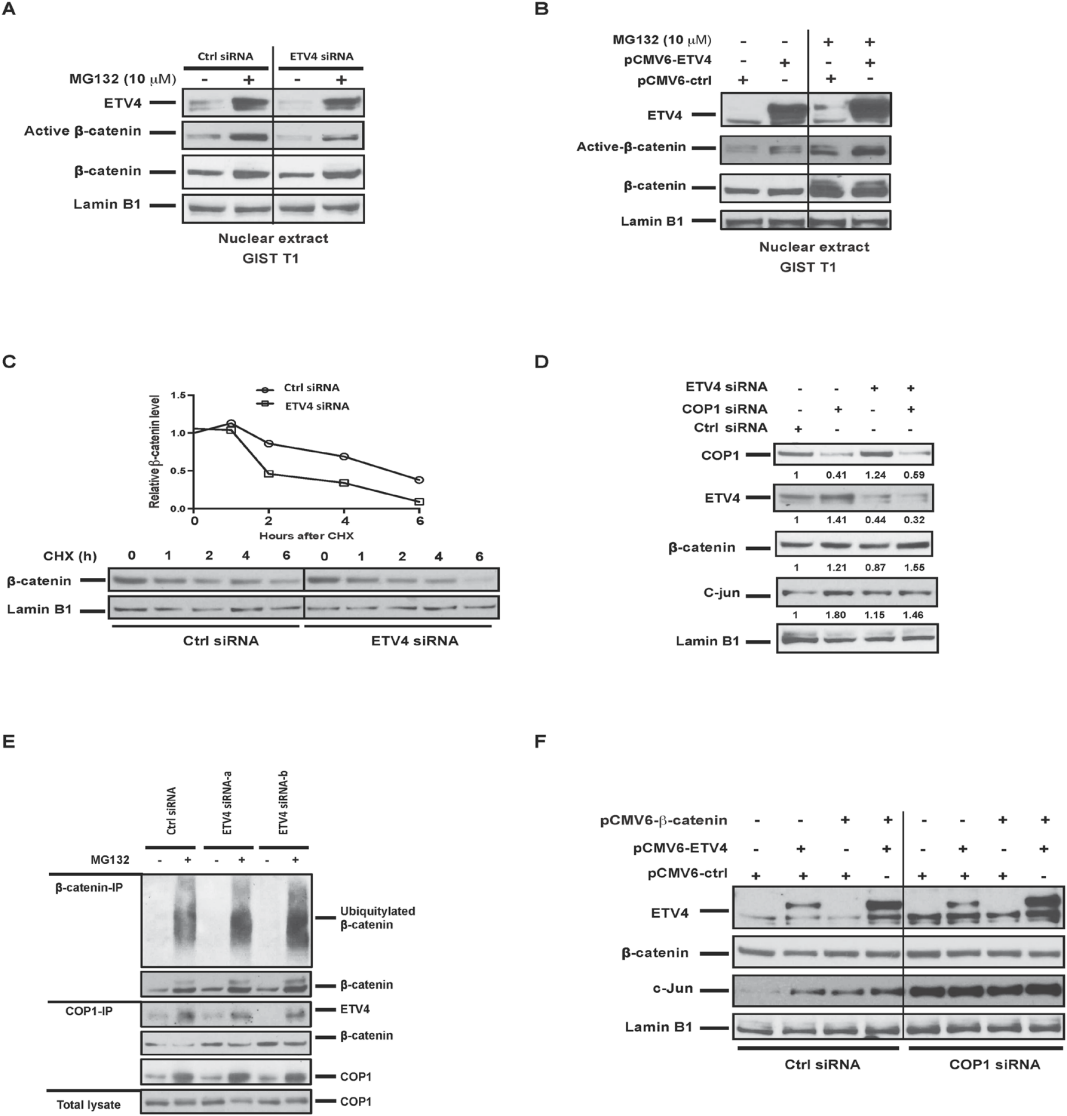
ETV4 interacts with COP1 to regulate nuclear β-catenin stability **(A)** GIST T1 cells were transfected with either ETV4 siRNA or control siRNA for 72h, and then cells were treated with MG132 10 μM for 6h prior to harvest. Nuclear protein extracts were analyzed by western blot as indicated. **(B)** GIST T1 cells were transfected with control or human ETV4 plasmids for 48h and treated with MG132 10 μM overnight before harvesting. Nuclear protein extracts were analyzed by western blot as indicated. **(C)** Representative CHX-chase assays (of 2 performed) to determine the stability (half-life) of nuclear β-catenin in GIST T1 cells 48h after transfection with control siRNA or ETV4 siRNA. Cells were collected after the addition of 200 mg/ml CHX at the indicated time points. Relative nuclear β-catenin levels were determined by normalizing to the loading control (lamin B1) and then normalizing to the *t* = 0h control siRNA. Immunoblots of nuclear extracts are shown. **(D)** GIST T1 cells were transfected with the indicated siRNA and harvested 48h later and nuclear extracts were analyzed by western blot. **(E)** GIST T1 cells were transfected with control or ETV4 siRNA for 72h and then treated with MG132 10 μM for 6h prior to harvest. Nuclear extracts were immunoprecipitated by either anti-β-catenin or anti-COP1 and western blot was performed as indicated. **(F)** GIST T1 cells were transfected with the indicated constructs and harvested 48h later and nuclear extracts were analyzed by western blot.

ETS family members have been shown to regulate Wnt/β-catenin signaling by directly binding to the promoter of the co-transcription factor LEF1 or by controlling β-catenin stability [11, 27]. However, in GIST T1 and GIST882 cells, even though active and total β-catenin were decreased by ETV4 knockdown and increased by ETV4 overexpression (Figure 3D & 3E, Figure 4A & 4E), β-catenin and LEF1 mRNA were not altered (data not shown). Therefore, we postulated that ETV4 might modulate β-catenin protein stability in GIST. We first examined nuclear β-catenin, which is the hallmark for Wnt/β-catenin activation. Nuclear ETV4 and β-catenin are unstable due to ubiquitin-mediated proteasomal degradation [28, 29]. To determine the role of ubiquitin and the proteasome in regulating nuclear β-catenin in GIST, we treated GIST T1 cells with the proteasome inhibitor MG132 for 6h. In cells treated with control siRNA and the proteasome inhibitor MG132, nuclear ETV4 and β-catenin were both stabilized. In cells treated with ETV4 SMARTpool siRNA, MG132 only partially restored the accumulation of active β-catenin, indicating that ETV4 knockdown resulted in active β-catenin destabilization (Figure 5A, lane 3 and 4). In contrast, the level of active β-catenin was increased in ETV4-overexpressing cells compared to control in GIST T1, and MG132 amplified the effect (Figure 5B). Thus, ETV4 regulated β-catenin signaling, at least partially through modulating nuclear β-catenin stability.

To further assess whether nuclear β-catenin stability is affected by ETV4, we examined nuclear β-catenin turnover in control or ETV4 siRNA-transfected GIST T1 cells in a cycloheximide (CHX) chase assay. ETV4 silencing significantly decreased the half-life of β-catenin (Figure 5C). In our previous study, the E3 ubiquitin ligase COP1 promoted nuclear β-catenin degradation [19]. Meanwhile, COP1 is known to bind to ETV4 and mediate its degradation [30]. COP1 knockdown resulted in ETV4 and β-catenin stabilization, as expected (Figure 5D). COP1 knockdown also abrogated β-catenin destabilization after ETV4 silencing, demonstrating that COP1 was required for the effects of ETV4 on β-catenin stability. We also observed slightly increased nuclear accumulation of COP1 by ETV4 knockdown, which facilitated β-catenin degradation (Figure 5D). Consistently, we also detected increased ubiquitination of nuclear β-catenin in ETV4-deficient cells compared to control cells by β-catenin co-immunoprecipitation in the presence of MG132 (Figure 5E, top panel). Meanwhile, interaction among endogenous COP1, ETV4, and β-catenin was detected by COP1 immunoprecipitation assays (Figure 5E, middle panel). Knockdown of ETV4 also facilitated the association of β-catenin with COP1 compared to control cells (Figure 5E, middle panel). Lastly, we demonstrated that ectopic expression of ETV4 and β-catenin together facilitated the stability of both proteins and cooperated to increase c-jun signaling, which was also COP1-dependent (Figure 5F). Overall, these data suggested that ETV4 partially contributes to β-catenin stability and interacts with β-catenin to drive oncogenic functions involved in GIST malignancy.

### ETV4 upregulation is associated with canonical Wnt activation in human GISTs

**Figure 6:**
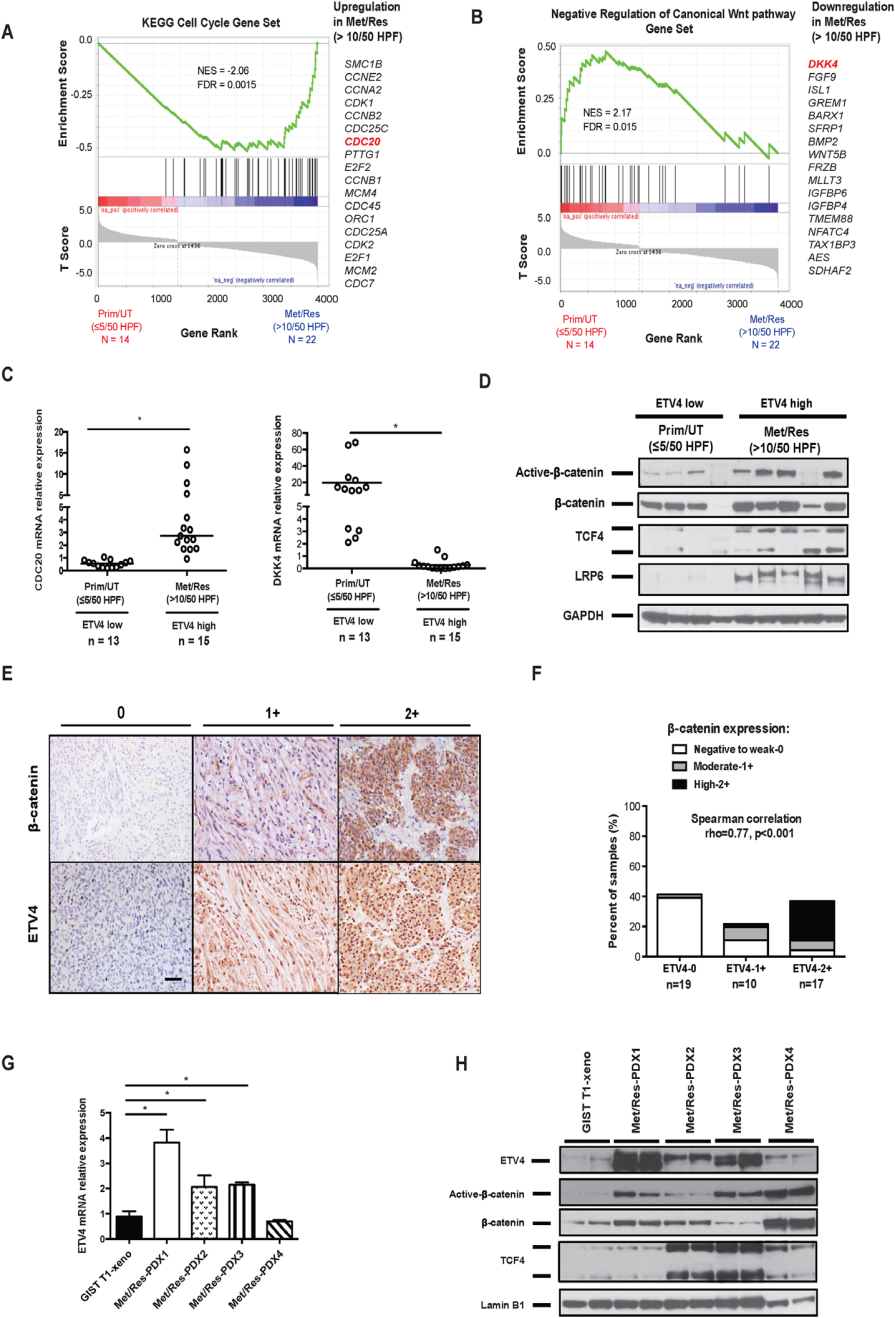
ETV4 upregulation is associated with canonical Wnt activation in human GISTs GSEA was performed on high throughput RNA sequencing data from 36 selected human GISTs. **(A)** Enrichment of cell cycle genes and **(B)** negative regulation of Wnt genes was found in Met/Res (>10/50 HPF) GISTs (N = 22) compared to Prim/UT (<5/50 HPF) GISTs (N = 14). False Discovery Rate (FDR) and Normalized Enrichment Score (NES) were indicated. **(C)** The relative mRNA expression of CDC20 and DKK4 was analyzed by real-time PCR in ETV4-low and high GIST tumors. Horizontal bars represent the median. Prim/UT (<5/50 HPF) – primary, untreated GIST (n = 13), Met/Res (>5/50 HPF) – metastatic, imatinib-resistant GIST (n = 15). Student’s *t* test; ^*^P < 0.05. **(D)** Western blot of Wnt pathway components in ETV4-high (metastatic, imatinib-resistant GISTs (Met/Res) with a mitotic rate >10/50 HPF) or ETV4-low (primary, untreated GISTs (Prim/UT) with a mitotic rate ≤5/50 HPF). **(E)** Representative staining of β-catenin and ETV4 expression in 46 GIST specimens. Staining intensity was scored as negative to weak (0), moderate (1+), or high (2+). Scale bar, 20 μm. **(F)** Correlation of ETV4 and β-catenin expression staining in 46 GISTs. Spearman’s rho = 0.77 with *P* = 0.001 per 2-tailed *t* test. **(G)** Real-time PCR showing relative mRNA expression of ETV4 in four PDXs compared to GIST T1 xenografts. All bars, means ± SEM. Student’s *t* test; ^*^P < 0.05. **(H)** Immunoblots of nuclear extracts from GIST T1 xenografts and four PDXs. PDX-1: *KIT* exon 11/13 mutation; PDX-2: WT *KIT* with *SDHB/KRAS* mutation; PDX-3: *KIT* exon 9/11 mutation; PDX-4: *KIT* exon 11 mutation with *CDKN2A* deletion. All four PDXs are from tumors of metastatic, imatinib-resistant GIST patients with a high mitotic rate.

To determine the relevance of our ETV4 findings in GIST cell lines, 36 selected human GISTs ((Prim/UT, ≤ 5/50 HPF, N = 12) vs. (Met/Res, >10/50 HPF, N = 22)) were analyzed by high throughput RNA-sequencing. Gene Set Enrichment Analysis (GSEA) was performed to probe the pathway differences between these two groups. Similar to our findings in GIST T1 cells, the results showed upregulation of cell cycle genes and activation of the canonical Wnt pathway in metastatic, imatinib-resistant GIST with a high mitotic rate (Figure 6A and 6B). Previously, we identified similar genes and pathways that might drive GIST tumor malignancy by transcriptome analysis of KIT^+^ cells isolated from 4 metastatic, imatinib-resistant tumors and 8 primary, untreated tumors [19]. We validated the *CDC20* findings in ETV4-low and ETV4-high human GISTs (Figure 6C, left). Dickkopf 4 (*DKK4*), as we have demonstrated before as a negative regulator of the Wnt pathway in GIST [19], was also lower in ETV4-high (metastatic, imatinib-resistant tumors with a mitotic rate >10/50 HPF) compared to ETV4-low (primary, untreated tumors with a mitotic rate ≤5/50 HPF) human GISTs (Figure 6C, right). In addition, we examined multiple Wnt/β-catenin pathway components in ETV4-high and ETV4-low human GISTs. Several key components of the canonical Wnt pathway were significantly upregulated in ETV4-high GISTs (Figure 6D). These included active β-catenin, TCF4, a key transcription factor that mediates the downstream effects of Wnt signaling, and low-density lipoprotein receptor-related protein (LRP6), which functions as a co-receptor required for canonical Wnt signaling [23]. These results demonstrated that the canonical Wnt pathway was upregulated in a subset of human GISTs, particularly those that are mitotically active with high ETV4 expression.

To directly examine the correlation between ETV4 and Wnt signaling in human GISTs, we stained 46 human GIST specimens for ETV4 and β-catenin. Both ETV4 and β-catenin staining density were scored as weak (0), moderate (1+), or high (2+), and varied among specimens (Figure 6E). There was a significant association (Spearman correlation rho = 0.77, *P* < 0.001) between ETV4 and β-catenin expression (Figure 6F). Previously, we showed that a greater percentage of metastatic, imatinib-resistant specimens had high β-catenin staining [19], just as we found here with ETV4. We also validated our preliminary findings using patient-derived xenografts (PDXs). We freshly isolated bulk tumor cells from four patients with metastatic disease and high ETV4 expression, and injected the cells into the flanks of NSG mice. Three of four PDXs had much higher levels of ETV4 mRNA and protein compared to GIST T1 xenografts (Figure 6G & 6H). Consistently, nuclear active β-catenin, β-catenin, and TCF4 were significantly higher in those PDXs compared to GIST T1 xenografts, indicating enhanced activation of Wnt/β-catenin signaling (Figure 6H). Thus, high levels of ETV4 expression contributed to tumor malignancy, as did β-catenin.

## DISCUSSION

Although the ETS transcription factors ETV1 and ETV4 exhibit highly conserved homology within their DNA-binding domains, they seem to have distinct roles in GIST. ETV1 appears to be a lineage survival factor in GIST as it is involved in tumor initiation and proliferation [13, 14]. Accordingly, we found that ETV1 mRNA expression was consistently high in human GISTs and did not differ between primary and metastatic tumors. Meanwhile, ETV4 mRNA expression was variable and correlated with tumor aggressiveness. While we did not see differences in ETV4 expression by tumor site (gastric versus small bowel) or mutation status (PDGFRa versus Kit), though the sample sizes were small for meaningful comparisons, most low-risk, human, primary, untreated GISTs had minimal or undetectable ETV4 expression. In contrast, there was a striking upregulation (> 5 fold) of ETV4 in mitotically active, primary, untreated GISTs and metastatic, imatinib-resistant GISTs with a high mitotic rate. Previously, we reported that after resection of primary GIST, tumor mitotic rate correlated with recurrence-free survival on multivariate analysis [31]. ETV4 overexpression has been associated with poor survival and metastasis in a variety of other tumors [18, 32]. In gastric cancer, the level of ETV4 mRNA correlated significantly with tumor invasiveness and recurrence, while ETV1 and ETV5 expression were not related to survival [33].

We found that ETV4 is an important oncogenic contributor to GIST since knockdown of ETV4 expression by RNA interference in both human and murine GIST cell lines suppressed tumor cell proliferation and invasion *in vitro*, as well as tumor growth *in vivo*. ETV4 is known to activate the transcription of multiple tumor-associated genes, including *MMP* genes, *Cox2*, *Cyclin D3*, and *Snail*, suggesting a role in tumor progression and metastasis [34]. Transcriptome analysis after ETV4 knockdown in GIST T1 cells showed a decrease in cell cycle genes. This observation is consistent with reports from other cancers, in which the target genes of ETV4 activation were highly enriched for cell cycle or cell growth functions. For example, ETV4 directly regulates *MYC* and other proliferation genes in prostate cancer cell lines [35]. ETV4 inhibition decreasing tumor cell invasion is consistent with ETV4 overexpression promoting invasion and metastatic potential via increased MMP expression and epithelial-to-mesenchymal transition (EMT) in other tumors [32, 36]. Accordingly, our array data in GIST T1 cells indicated that ETV4 knockdown significantly affected the Wnt signaling pathway. Those data prompted us to further investigate the relationship of ETV4 and components of the Wnt/β-catenin pathway in GIST. Indeed, ETV4-high human GISTs had cytoplasmic and/or cytoplasmic/nuclear β-catenin staining, which are hallmarks of aberrant Wnt/β-catenin signaling. Our previous findings showed that activation of the canonical Wnt pathway and accumulation of nuclear active β-catenin were present in a subset of human GISTs [19]. Furthermore, in our PDX models, high ETV4 expression was also associated with Wnt/β-catenin activation.

Our GSEA data linked ETV4 expression with Wnt pathway deregulation in metastatic, imatinib-resistant human GIST. It is important to note that since most metastatic, imatinib-resistant patients were also recurrent GISTs in our cohort, we could not analyze these groups separately although recent literature suggests there may be significant differences between these two groups [37]. Nevertheless, there are several potential mechanisms to explain the interaction of ETV4 and Wnt/β-catenin signaling in GIST. First, although ETV4 knockdown did not alter β-catenin mRNA, it did affect β-catenin protein levels. While Wnt/β-catenin can be activated in numerous ways in cancer, nuclear β-catenin stabilization is critical for its final activation and oncogenic function. Recently, several E3 ligases have been shown to regulate transcriptionally active nuclear β-catenin in certain tumor types [28, 38]. We previously demonstrated that COP1 could regulate nuclear β-catenin stability in GIST cell lines [19]. In the present study, we also identified COP1 as a negative regulator of ETV4. Knockdown of ETV4 facilitated β-catenin ubiquitination and degradation via COP1. Ectopic expression of ETV4 further potentiated active β-catenin signaling in the presence or absence of Wnt3a or when β-catenin was overexpressed. Meanwhile, overexpression of ETV4 and β-catenin increased the stability of both proteins, as well as c-jun signaling, which has been reported to stabilize β-catenin [26]. Collectively, it is reasonable to consider that ETV4 enhanced nuclear β-catenin stability through inhibiting β-catenin protein degradation, thereby driving GIST malignancy.

Another potential mechanism to explain the relationship of ETV4 and β-catenin is that ETV4 is associated with the expression of pathway components and regulators of Wnt/β-catenin signaling. ETV4-high human GISTs had increased pathway activation compared to ETV4-low tumors, as evidenced by dephosphorylated active β-catenin, the transcription factor TCF4, and the co-receptor LRP6. Moreover, the Wnt pathway antagonist DKK4 was substantially lower in ETV4-high human GISTs. Our previous work showed that silencing of DKK4 in GISTs contributed to aberrant Wnt pathway activation [19].

An additional link between ETV4 and Wnt/β-catenin signaling is that ETV4 knockdown decreased phosphorylated GSK3β. It is well known that phosphorylation of GSK3β serine 9 is regulated by the PI3K/AKT signaling pathway. Overexpression of phosphorylated GSK3β serine 9, coupled with active β-catenin, has been associated with poor prognosis in various cancers [39]. Other data have shown that expression and activation of ETV4 is correlated with activation of PI3K signaling in tumor progression [18], and ETV4 knockdown resulted in a downregulation of phospho-AKT [40]. In GIST, PI3K/Akt is constitutively activated, which promotes phosphorylation and inactivation of GSK3β, allowing stabilization of the β-catenin destruction complex. Previously, we showed that inhibition of KIT by imatinib reduced AKT activation, as well as ETV4 expression in mouse *Kit*^*V558Δ/+*^ tumors [16]. As a feedback circuit, *in vitro* knockdown of ETV4 significantly decreased activated AKT signaling with a concomitant decrease of phospho-GSK3β in GIST T1, GIST882, and murine S2 GIST cell lines. In contrast, overexpression of ETV4 in GIST cell lines increased phospho-AKT activation and active β-catenin. A specific dual PI3K/AKT inhibitor, NVP-BEZ235, also inhibited GSK3β phosphorylation and β-catenin accumulation in the presence or absence of Wnt pathway activators in GIST cells (Supplementary Figure 1). Constitutive activation of PI3K/AKT signaling may inhibit GSK3β and stabilize β-catenin, thereby contributing to tumorigenesis [41]. Several studies have shown that in other tumors, ETS family members collaborated with other signaling pathways to induce tumorigenesis and invasive behavior [17]. We also found that knockdown of ETV4 by siRNA can disrupt activation of phospho-ERK signaling. Similar to ETV1, treatment with a MAP kinase inhibitor (PD0325901) also decreased ETV4 expression (unpublished data), suggesting that MAPK/ERK may be one of the multiple players regulating ETV4 expression and its association with Wnt signaling in GIST. It is plausible that ETV4 may also converge with Wnt signaling through the AKT/GSK3β signaling cascades or other signaling cascades in GIST cell lines. Simultaneous activation of ETV4 and β-catenin signaling may lead to more aggressive phenotypes through elevated expression of Wnt/β-catenin signaling components.

Thus, we have shown that ETV4 plays a critical role both *in vitro* and *in vivo* in mouse and human GIST. ETV4 expression modulated the cell cycle genes and Wnt/β-catenin signaling and was associated with an aggressive phenotype in human GIST. ETV4 also promoted tumor malignancy by stabilizing nuclear β-catenin, and synergizing with the PI3K/Akt pathway to drive activation of Wnt/β-catenin. Knockdown of ETV4 effectively suppressed GIST tumor growth. The synergistic impact of ETV4 and β-catenin signaling contributes to GIST tumorigenicity and aggressiveness and offers new therapeutic approaches.

## MATERIALS AND METHODS

### Human tumor samples

Under an IRB approved protocol, we obtained 55 GIST specimens from consenting patients undergoing tumor resection at Memorial Sloan Kettering Cancer Center. Mitotic rate (number of mitoses per 50 high power fields) and mutation status for each specimen were obtained from pathology reports. Tumors were categorized into 2 groups based on the type of tumor (primary or metastatic) and the radiologic tumor response to tyrosine kinase inhibitors at the time of surgery (untreated or resistant (i.e., progressing despite tyrosine kinase inhibition)). Single cell suspensions were generated from fresh tumor tissues by a modified collagenase method as before [21], and separated into KIT^+^ (i.e., tumor cells) and KIT^-^ populations using human CD117 microbeads (Miltenyi Biotec). The purity of KIT^+^ cells was greater than 90% by flow cytometry.

### Immunohistochemistry

Formalin-fixed and paraffin-embedded human GISTs were sectioned at 5 μm thickness. Antigen retrieval was achieved with citrate buffer. Immunohistochemistry was performed using anti-human ETV4 IgG (ab74045, Abcam) and anti-human β-catenin IgG (Clone β-catenin 1, DAKO) or the corresponding isotype control IgG (DAKO).

### RNA interference

The human GIST882 cell line (*KIT* exon 13 mutant) as well as human GIST T1 and murine S2 GIST cells (both *KIT* exon 11 mutant) have been described previously [7, 21]. For stable ETV4 knockdown, GIST882 cells or murine S2 cells were transfected with two specific ETV4 SMARTvector lentiviral shRNAs or SMARTvector non-target controls (all from Thermo Scientific). Transduced cells were selected with 5 μg/ml puromycin (Sigma-Aldrich) 2 days later and stable cell lines were established when all cells expressed GFP. For transient gene knockdown, GIST T1 cells were transfected with 30 nM of ON-TARGETplus siRNA specific for human ETV4 (J-004207-05, J-004207-06, J-004207-07), ON-TARGETplus SMARTpool siRNA for human ETV4 (L-004207-00), ON-TARGETplus SMARTpool siRNA for human ETV1 (L-003801-00), ON-TARGETplus SMARTpool siRNA for human COP1 (E-007949-00), or non-target control siRNA (D-001810-10-05) (Thermo Scientific) using Lipofectamine RNAiMAX (Invitrogen) for 48, 72, or 96h. To generate cells transiently overexpressing ETV4, GIST T1 or GIST882 cells were transfected with either control plasmid (pCMV6-ctrl, PS10001, Origene) or human ETV4 plasmid (pCMV6-ETV4, RC202010, Origene) using Lipofectamine 2000 (Invitrogen) according to the manufacturer’s protocol.

### Cell viability, invasion assays, and *in vivo* tumor growth

Cell viability (Cell Counting Kit 8, Dojindo) of the stable GIST882 or murine S2 cells with control shRNA or ETV4 shRNA in 96-well plates was determined at 48 and 72h according to manufacturer’s instructions by optical density at 450 nm. Cell invasion of 1x10^5^ GIST T1, stably transduced GIST882, or murine S2 cells in 0.5 ml of serum-free medium was determined by placing them above inserts in a 24-well Matrigel invasion chamber (Becton Dickinson). Wells contained 15% FCS growth medium to serve as a chemoattractant. After 24h, the non-invading cells were gently removed from the upper chamber by a cotton swab. Cells that invaded into the lower chamber were fixed and stained with Diff-Quick (Siemens). The number of invading cells in the 2 groups was compared according to the manufacturer’s instruction. To determine the effect of ETV4 knockdown on tumor growth *in vivo*, 1x10^5^ stably transfected GIST882 cells or murine S2 cells were injected subcutaneously (s.q.) into the right flank of NOD.Cg-Prkdc^scid^IL12rg^tm1Wjl^/SzJ (NOD scid gamma (NSG), Jackson Laboratory) mice. All mouse studies were approved by the Institutional Animal Care and Use Committee.

### Real-time PCR and microarray analysis

Total RNA was extracted from human GIST tissues or transfected cells using the RNAeasy Plus Mini Kit (Qiagen). Real-time PCR was performed as before [7]. The following primers were used: human ETV4 (Hs00385910_ml; Hs00383361-g1), ETV1 (Hs00951941_ml), CDKN1C (Hs00175938_ml), SFRP4 (Hs00180066_ml), DKK4 (Hs00205290_ml), and GAPDH (Applied Biosystems). Microarray was performed in the Integrated Genomics Core Laboratory of Sloan Kettering Institute using Human Genome U133A 2.0 microarrays (Affymetrix). Microarray data were analyzed using Partek Genomics Suite version 6.5. After log transformation and quantile normalization, ANOVA was performed to compare multiple groups. Statistically significant genes with a False Discovery Rate <0.05 were selected and analyzed using KEGG Pathway Analysis software (Ingenuity Systems).

### Western blot

Western blot of whole protein lysates or nuclear proteins from frozen tumor tissues or cells was performed as previously described [21]. Antibodies for cyclin D1 (92G2), β-catenin (6B3), active β-catenin (S33/S37/T41), TCF4 (C48H11), LRP6 (C47E12), phospho-GSK3β (Ser9) (5B3), GSK3β (27C10), c-Jun (60A8), phospho-AKT (D9E), alpha-tubulin (DM1A), and GAPDH (D16H11) were purchased from Cell Signaling Technology. Other antibodies included anti-human ETV4 (SC-113, Santa Cruz), anti-human COP1 (ab56400, Abcam), lamin B1 (PA5-19468, Invitrogen), and anti-active β-catenin (Clone 8E7, Millipore). ImageJ software was used to measure the relative density for signaling expression.

### Cycloheximide (CHX) chase and proteasome inhibition assays

GIST T1 cells were transiently transfected with control siRNA or ETV4 SMARTpool siRNA for 48h. Cells were collected after the addition of 200 μg/ml CHX at the indicated time points with or without 10 μM MG132. Cell lysates from nuclear extracts were assessed for signaling. Quantification was achieved by Image J software. The final β-catenin turnover rate at each time point is the percentage of β-catenin/lamin B1, then expressed relative to the *t* = 0h negative siRNA control.

### Cell cycle analysis

GIST T1 cells were transiently transfected with neg siRNA and ETV4 siRNA for 48 hours. Cells were harvested, washed in PBS and fixed with ice-cold 70% ethanol at 4°C for at least 30 min. After washing with PBS, cells were incubated with propidium iodide (PI) (0.05 mg/ml) and ribonuclease (Sigma) at 37°C for 30 min. Flow cytometry was performed for cell cycle distribution.

### Gene set enrichment analysis (GSEA)

RNA was isolated from 36 selected human GIST specimens using the RNAeasy Plus Mini Kit (Qiagen). High throughput RNA sequencing was performed by the Integrated Genomics Core laboratory of Sloan Kettering Institute using the Illumina platform. GSEA was performed on the RNA sequencing data using gene sets from the MSigDB (Molecular Signatures Database) using the GSEA software package (http://software.broadinstitute.org/gsea) [42]. The gene sets showing a false discovery rate (FDR) of less than 0.25 were considered enriched between the classes under comparison.

### Patient derived xenografts (PDXs)

PDXs were established from 4 metastatic, imatinib-resistant human GISTs. Bulk tumor cells were freshly isolated from GIST tumors using collagenase as above and 1x10^6^ cells were injected into the flanks of NSG mice. Mutation status of each tumor was determined. GIST T1 xenografts were used as a comparison with these PDXs, since we have not been able to generate a PDX from a primary, untreated human GIST.

### Statistical analysis

Data are expressed as mean ± SEM or median. Differences were detected by unpaired 2-tailed Student’s *t* tests unless otherwise indicated using Prism 6.0 software (Graph Pad Software). P < 0.05 was considered significant.

## SUPPLEMENTARY MATERIALS FIGURE AND TABLES


