# Supplementary material for: ETV4 collaborates with Wnt/β-catenin signaling to alter cell cycle activity and promote tumor aggressiveness in gastrointestinal stromal tumor
Source: Oncotarget. 2017 Dec 11;8(69):114195–209. doi: 10.18632/oncotarget.23173 (PMC5768396; doi:10.18632/oncotarget.23173)
Supplement: Supplementary file 1 [file oncotarget-08-114195-s001.pdf]

ETV4 collaborates with Wnt/β-catenin signaling to alter cell cycle activity and promote tumor aggressiveness in gastrointestinal stromal tumor

SUPPLEMENTARY MATERIALS

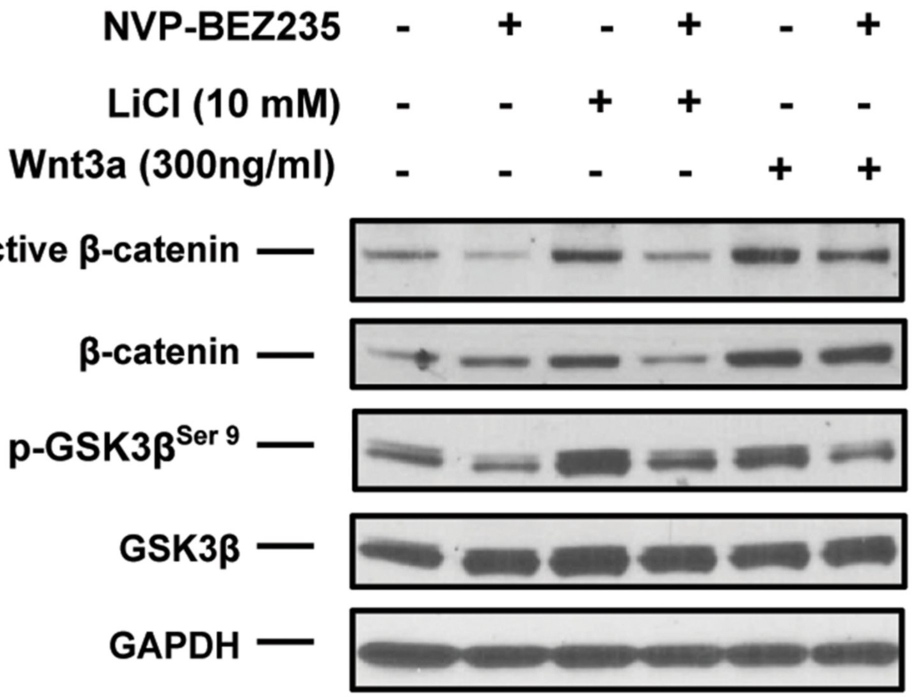

Supplementary Figure 1: Cross talk between PI3K/Akt and GSK3β contribute to β-catenin stability. GIST882 cells were pretreated with 500 nM NVP-BEZ235, a dual PI3K/Akt inhibitor for 2h, and then treated with 10 mM LiCl for 14h or 300 ng/ml rhWnt3a for 4h. Cell lysates were analyzed by Western blot with indicated antibodies.

Supplementary Table 1: Clinicopathologic characteristics of 55 specimens from 55 patients

	Primary/untreated	Metastatic/resistant
Number of patients	32	23
Number of specimens	32	23
Median age (years)	41 (38-85)	56 (39-73)
Female	11	6
Male	21	17
Primary	32	0
Metastatic	0	22
Local recurrence	0	1
Primary Location		
Stomach	29	5
Small intestine	1	7
Other	2	11
Tyrosine kinase inhibitor		
Imatinib	Not applicable	23
Sunitinib		6
Other		4
Treatment duration (months)	Not applicable	36-132
Mutational status		
KIT exon 9	0	3[a]
KIT exon 11	9	18[b]
KIT exon 13	0	7
PDGFRA	4	1
WT	1	1
Unavailable	18	0

[a]3 patients also had a secondary KIT exon 13 mutation.

[b]4 patients also had a secondary KIT exon 13 mutation, and 3 had a secondary exon 17 mutation.

Supplementary Table 2: Differentially regulated pathways in control siRNA vs. ETV4 siRNA knockdown

Rank	Pathway name	Input genes in pathway	Pathway genes on chip	Input gene name	P-value
1	Antigen processing and presentation	1	79		1.80E-03
2	DNA replication	3	33		2.96E-02
3	Parkinson's disease	3	104		3.20E-02
4	Cell cycle	5	105	CDKN1C, SMC1A, CDC23, GADD45A, ORC4	3.21E-02
5	Wnt signaling pathway	3	134	SFRP4, TBL1X, PPP2R1B	3.97E-02
6	Nucleotide excision repair	3	42		4.77E-02
